# Assessing the feasibility, theoretical interest, and observed behaviors of adding genetic data to a patient-reported multiple sclerosis registry

**DOI:** 10.1177/20552173261467203

**Published:** 2026-08-21

**Authors:** Kaarina Kowalec, Douglas Menendez, Gary Cutter, Robert J Fox, Ruth Ann Marrie, Amber Salter

**Affiliations:** 1 College of Pharmacy, Rady Faculty of Health Sciences, University of Manitoba, Winnipeg, MB, Canada; 2 Department of Medical Epidemiology & Biostatistics, Karolinska Institutet, Solna, Sweden; 3 Department of Biochemistry & Medical Genetics, Max Rady College of Medicine Rady Faculty of Health Sciences, University of Manitoba, Winnipeg, MB, Canada; 4 Department of Neurology, UT Southwestern, Dallas, TX, USA; 5 Department of Biostatistics, University of Alabama at Birmingham, Birmingham, AL, USA; 6 Mellen Multiple Sclerosis Center, Cleveland Clinic, Cleveland, OH, USA; 7 Department of Internal Medicine, Max Rady College of Medicine, Rady Faculty of Health Sciences, University of Manitoba, Winnipeg, MB, Canada; 8 Department of Medicine, Faculty of Medicine, Dalhousie University, Halifax, NS, Canada; 9 Nova Scotia Health, Halifax, NS, Canada

**Keywords:** Multiple sclerosis, genetics, registry, saliva samples, biospecimens

## Abstract

**Background and Objectives:**

We aimed to assess the feasibility and acceptability of self-collected saliva for genetics from participants of a registry and determine whether responses to hypothetical questions regarding sharing of genetic information reflected observed behaviors.

**Methods:**

We conducted a cross-sectional study collecting saliva for a genetic study in the North American Research Committee on MS (NARCOMS) registry. We linked participants to a prior survey for a subset of participants with responses, to examine factors associated with concordance between consent status and hypothetical willingness to share genetic information using logistic regression.

**Results:**

Of ∼6200 participants contacted, 1227 expressed interest in the genetic study, 763 consented (62.2% of interested, ∼10% of those contacted) and most returned saliva samples (>80% of those consented). We found low agreement between the genetic study response and willingness to link genetic data (adjusted kappa = 0.186, standard error = 0.029). Multivariable regression identified no factors significantly associated with concordance between consent status and hypothetical willingness to share genetic information, though findings may reflect limited power.

**Conclusion:**

Among participants who expressed interest, most were willing to provide saliva for genetics studies, though hypothetical sharing of genetic information did not reflect actual behaviors, and overall participation represented a minority of those contacted.

## Introduction

While our understanding of multiple sclerosis (MS) has evolved substantially, much remains unknown related to its long-term outcomes and their predictors. Registries such as the North American Research Committee on MS Registry (NARCOMS) collect longitudinal health-related data from individuals that can fill these knowledge gaps.^1^ NARCOMS and other registries have driven advances in MS research, including investigating comorbidity,^2^ racial and socioeconomic disparities in outcomes,^3^ such as disability, and caregiver mistreatment in MS.^4^

Sources of registry information vary by registry and could include clinician-reported data, performance-based data, or patient-reported outcomes. Linkage of registries to other data sources such as electronic health records offers the opportunity to answer different research questions in a cost-effective way. A recent review recommended that incorporating the collection of biospecimens into MS registries could further enhance impact.^5^ Impacts include advancing our knowledge of MS severity,^6^ or gene-by-environment interactions.^7^ Recently, interest has grown in understanding the contributions of genetic factors to MS outcomes, including MS disease activity and progression.^8,9^ Adding genetic data to long-term MS registries could facilitate the identification of novel genetic factors associated with disease outcomes.

A recent study involving 4980 participants in the NARCOMS registry (6998 invited, 71.2% response rate) suggested that three-quarters of 4662 eligible participants would be willing to agree to linkage of their administrative (health claims) data to support clinical trials (n = 3524/4662; 75.6%).^10^ However, a lower proportion (∼65%) were willing to link their genetic information, and it is unclear if the willingness to share genetic data would differ if the study were observational rather than a clinical trial. Willingness to link genetic data was greater among participants with higher levels of education and income, higher disability levels and prior trial participation. These response patterns form the basis of the hypothetical willingness categories used in the concordance analysis in the current study.

Therefore, we conducted a pilot study to assess the feasibility and acceptability of collecting saliva samples for eventual genomic data generation to the NARCOMS registry. Feasibility was defined by participation rates. We also sought to determine whether past responses to hypothetical questions regarding sharing of genetic information from the previous publication^10^ reflected observed behavior in the current pilot study, and what sociodemographic and clinical factors were associated with concordant versus discordant responses to the data linkage survey and the consent for genetic data collection. Concordance was defined as agreement between a participant's consent status in the current genetic study and their previously stated hypothetical willingness to share genetic data. Understanding whether hypothetical willingness reflects actual participation behavior is important because registries such as NARCOMS or other studies rely on survey-based estimates to project recruitment, allocate resources, and assess feasibility prior to launching genetic substudies. Discordance between stated and observed behavior would suggest that such estimates should be interpreted cautiously. We hypothesized that 70% of NARCOMS participants will be willing to provide saliva sample for genetics studies in MS based on a previous non-MS genetic study.^11^

## Methods

### Design

We designed a cross-sectional study to pilot recruitment, collection and return of saliva kits at-home among participants in the NARCOMS Registry. The NARCOMS Registry is a longitudinal registry for persons with MS. Registry participants provide demographic and clinical information electronically or on paper at enrollment and semi-annually thereafter.^1^ Extensive efforts have validated diagnoses of MS, its measures of symptom severity and disability (PDDS) in NARCOMS.^12–14^

### Sample collection

We conducted sample collection in two phases: an initial optimization phase followed by expanded recruitment. In the first phase, we distributed an invitation to participate in a genetics substudy via REDCap,^15^ the current method of NARCOMS survey administration to 995 NARCOMS participants. These participants were selected based on having a longer length of participation in the registry and reporting a willingness to contribute genetics for long term follow-up in the Fall 2023 survey.^10^ We distributed invitations five times to maximize response rates. During the second phase, the study flyer was sent directly to participants after completion of the semi-annual update survey and included in the *NARCOMS Now* magazine which is sent to all active participants (n ≈ 6200). After the subject expressed interest in study participation, the participant received a consent form to review, and the research team was available to answer questions. The participant had the option to consent online using the REDCap e-consent framework or have a paper consent form with a postage-paid return envelope mailed to them. Sample selection was enriched for participants with the longest NARCOMS follow-up time as this would permit greater ability to investigate longitudinal outcomes in the future. Upon completion of the consent form, a saliva collection kit (DNA Genotek OG-500, Ottawa, Canada), instructions, and postage-paid return envelope was mailed to participants.

### Data linkage

As described previously,^10^ NARCOMS participants reported their perspectives about genetic and nongenetic data linkage in clinical trials in Fall 2023 using questions adapted from a Canadian feasibility study in a lymphoma population.^16^ As part of that survey, participants were advised that they had hypothetically agreed to clinical trial participation, and were asked if they were willing to allow specific data elements to be stored at a central research coordinating site. Genetic data were among the specific data elements queried where potential responses were “yes, store centrally to help with analyses”; “perhaps, depending on circumstances it should be fine to store centrally”; “no, I will not want it to be stored centrally”; “unsure or undecided”; and “prefer not to answer”.

### Covariates

Each enrollment questionnaire captures information about birthdate, race, ethnicity, educational attainment, sex and age of MS symptom onset. The Fall 2023 questionnaire provided updated information related to annual household income, health insurance, employment, marital status, comorbidities conditions, health behaviors, severity of disability, and US zip code. Race was grouped as White, African American/Black, and other; the number of participants reporting Hispanic ethnicity was too small for analysis (<50). We dichotomized educational attainment as less than or equal to high school/General Educational Development Test, and postsecondary (associate's degree, bachelor's degree, postgraduate education, and technical degree). Annual household income (in US dollars) was categorized as <$50,000 [reference], $50,001–$100,000, >$100,000, and “I do not wish to answer.” We dichotomized marital status as single (never married/separated/divorced/widowed) or partnered (married/co-habiting). Health insurance was dichotomized as yes versus no. Employment status was reported as full-time, part-time or not employed. Participants reported comorbidities that had been diagnosed by a physician, including anxiety disorder, depression, autoimmune thyroid disease, diabetes, hypertension, hyperlipidemia, heart disease, chronic lung disease, irritable bowel syndrome, psoriasis, fibromyalgia, sleep apnea, stroke, and cancer. The physical comorbidities were grouped as a count (0, 1, 2, ≥3) and psychiatric comorbidities were dichotomized as any versus none. Current smoking status, any physical activity, and alcohol intake were categorized as (yes vs. no). Participants reported their disability using Patient Determined Disease Steps (PDDS), which includes one item with nine potential responses, where 0 is normal and 8 is bedridden.^17^ PDDS correlates strongly with the physician-scored Expanded Disability Status Scale and is reliable.^10^ Consistent with prior work, we categorized the PDDS as mild (0–1), moderate (2–4), and severe (5–8).^11^ To obtain area-level socioeconomic information we used the area level deprivation index (ADI),^12,13^ which is based on data regarding education, employment, housing quality, and poverty drawn from the 2020 American Community Survey. ADIs are provided in percentile rankings from 0 (lowest disadvantage) to 100 (highest disadvantage) and we grouped them into quartiles.

### Statistical analysis

The response status of each participant approached for consent was categorized as a nonresponder, responder who declined consent, or responder who consented. For each participant who consented, we report the frequency (percent) who ultimately returned a saliva kit and how long this took after the kit was distributed. Using descriptive statistics including mean (standard deviation [SD]), median (interquartile range [IQR]) and frequency (percent), we characterized the study sample overall and according to response status. We used student's t-tests, Wilcoxon or Kruskal–Wallis tests, and chi-square tests for bivariate analyses.

We linked data for participants who were approached about the genetics substudy to their responses from the Fall 2023 survey. We used a simple and prevalence- and bias-adjusted kappa statistic with 95% confidence interval (95% CI) to assess agreement between genetic study response status (consented vs. not) and willingness to link genetic data (yes or perhaps vs. no or unsure).

We also examined factors associated with concordance between consent status and hypothetical willingness to share genetic information using a binary logistic regression model, where discordance = 0, and concordance = 1, including factors associated with concordance in the bivariate analyses (*p* < 0.05). Based on our prior study, variables considered included: age, sex (female as reference), race (White as reference), education (high school as reference), income (<$50,000 as reference), marital status (single as reference), employment status (full-time as reference), neighborhood deprivation in quartiles (lowest as reference), comorbidities (0 as reference), smoking status (no as reference), physical activity (no as reference), alcohol intake (no as reference), disability status (mild as reference), disease duration (continuous), and prior clinical trial participation (no as reference).

Statistical analyses were conducted using SAS V9.4 (SAS Institute Inc., Cary, NC, USA). P-values <0.05 were deemed statistically significant.

## Results

**Figure 1.**
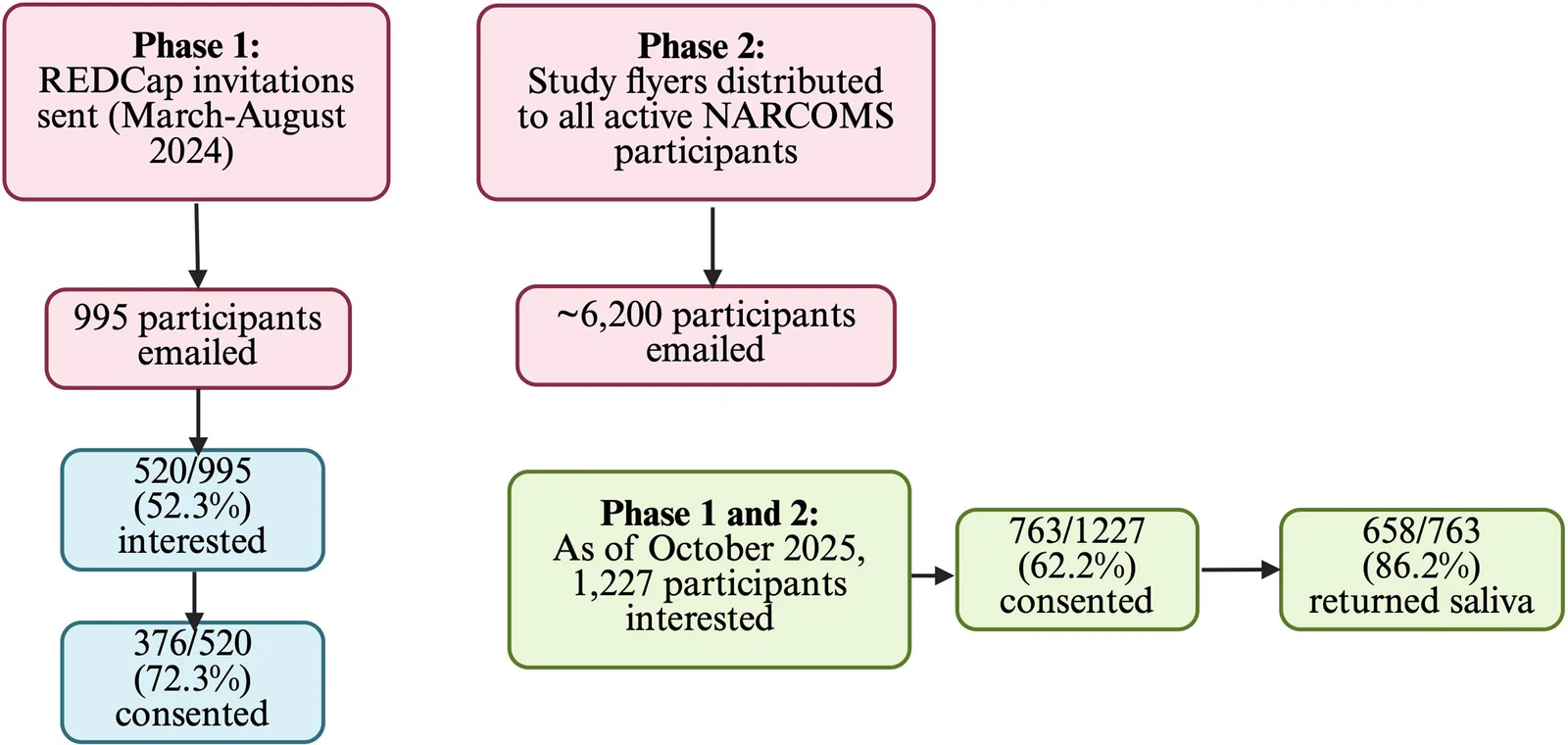
Flow chart of participant recruitment from NARCOMS registry for genetic study. Created in https://BioRender.com.

Participant flow across both recruitment phases is summarized in Figure 1; key numbers are described below. The first phase of recruitment began March 2024 and ended August 2024. A total of 995 participants were directly emailed for study participation and 520 (52.3%) participants contacted NARCOMS with interest in participating. Of those that reached out, 376 (72.3%) consented. A second phase of recruitment included sending ∼6200 participants study flyers. Following both recruitment phases, as of October 2025, 1227 participants contacted NARCOMS about participation and 763 (62.2%) consented with 658 returning a saliva sample (86.2%). Of the 763 consenting, majority consented using the REDCap e-consent framework (n = 554, 72.6%) compared to receiving a paper consent form (n = 209, 27.4%). Median days to receive returned collected sample via prepaid post was 17 days (interquartile range: 14, 24 days).

Overall, 4662 participants responded to the Fall 2023 semiannual update survey (Table 1). Of the 1146 participants who responded, 699 consented and 447 declined to consent. Those who consented to participate in the genetics substudy were more likely to have higher annual household income, more likely to be partnered, employed, more likely to be seen by an MS specialist or have a progressive MS course, compared to those who declined (Table 1).

**Table 1.** Characteristics of NARCOMS participants from the Fall 2023 survey based on their consent status for the genetic substudy.

Characteristics	Overall	(1) Nonresponder^a^	(2) Declined	(3) Consented	*P*-value
N	4662	3516	447	699	—
Age at time of fall 2023 survey (years), mean (SD)	66.1 (9.74)	66.4 (9.78)	65.8 (9.13)	64.5 (9.76)	<.001
Age at MS symptom onset (years), mean (SD)	31.4 (9.91)	31.4 (9.92)	31.5 (9.03)	31.6 (10.36)	0.828
Age at MS diagnosis (years), mean (SD)	38.9 (9.84)	39.0 (9.87)	39.3 (9.02)	38.4 (10.14)	0.247
Disease duration (years), mean (SD)	26.1 (9.73)	26.4 (9.89)	25.5 (8.83)	25.2 (9.42)	0.003
Clinical course, n (%)					0.005
CIS/relapsing remitting	2492 (54.9)	1846 (54.0)	256 (58.7)	390 (57.0)	
Primary or secondary progressive	1665 (36.7)	1255 (36.7)	154 (35.3)	256 (37.4)	
Don’t know	379 (8.4)	315 (9.2)	26 (6.0)	38 (5.6)	
Female, n (%)	3788 (81.3)	2841 (80.8)	370 (82.8)	577 (82.5)	0.384
Race, n (%)					0.048
White	4079 (87.5)	3089 (87.9)	383 (85.7)	607 (86.8)	
Black	104 (2.2)	87 (2.5)	8 (1.8)	9 (1.3)	
Other	479 (10.3)	340 (9.7)	56 (12.5)	83 (11.9)	
Education at enrollment, n (%)					<.001
≤High school/GED	1855 (40.7)	1513 (44.1)	126 (28.7)	216 (31.4)	
≥Postsecondary	2703 (59.3)	1919 (55.9)	313 (71.3)	471 (68.6)	
Annual household income, n (%)					<.001
Less than $50,000	1331 (28.8)	1049 (30.2)	125 (28.0)	157 (22.5)	
$50,001-$100,000	1185 (25.7)	837 (24.1)	128 (28.7)	220 (31.6)	
Over $100,000	1038 (22.5)	688 (19.8)	120 (26.9)	230 (33.0)	
I do not wish to answer	1065 (23.1)	902 (25.9)	73 (16.4)	90 (12.9)	
Any health insurance, n (%)	4474 (96.0)	3355 (95.4)	429 (96.0)	690 (98.7)	<.001
Marital status, n (%)					0.011
Single	1521 (32.7)	1184 (33.8)	141 (31.8)	196 (28.0)	
Partnered	3125 (67.3)	2319 (66.2)	303 (68.2)	503 (72.0)	
Employment status, n (%)					<.001
Full-time	786 (16.9)	564 (16.1)	78 (17.5)	144 (20.6)	
Part-time	395 (8.5)	269 (7.7)	41 (9.2)	85 (12.2)	
Not employed	3474 (74.6)	2677 (76.3)	327 (73.3)	470 (67.2)	
Rural-urban commuting area, n (%)					0.545
Urban-focused	3935 (85.6)	2964 (85.9)	370 (83.0)	601 (86.0)	
Large rural city/town-focused	361 (7.9)	269 (7.8)	40 (9.0)	52 (7.4)	
Small or isolated rural town-focused	301 (6.5)	219 (6.3)	36 (8.1)	46 (6.6)	
Area deprivation index, mean (SD)					
National rank	39.8 (24.85)	40.4 (24.97)	39.4 (24.21)	37.4 (24.51)	0.013
State rank	4.4 (2.66)	4.5 (2.67)	4.2 (2.62)	4.1 (2.65)	0.002
Area deprivation index, national rank (quantiles), n (%)					0.103
0–25	1546 (34.0)	1132 (33.1)	149 (33.4)	265 (38.4)	
26–50	1531 (33.6)	1146 (33.5)	157 (35.2)	228 (33.0)	
51–75	994 (21.8)	759 (22.2)	99 (22.2)	136 (19.7)	
76–100	482 (10.6)	380 (11.1)	41 (9.2)	61 (8.8)	
Source of care, n (%)					<.001
General practitioner	282 (6.1)	241 (6.9)	19 (4.3)	22 (3.2)	
Private neurologist	2173 (46.9)	1658 (47.5)	202 (45.2)	313 (45.0)	
Specialized MS center	1691 (36.5)	1198 (34.3)	190 (42.5)	303 (43.6)	
Veteran's administration	149 (3.2)	116 (3.3)	12 (2.7)	21 (3.0)	
Other	72 (1.6)	57 (1.6)	5 (1.1)	10 (1.4)	
Do not receive care for MS	269 (5.8)	224 (6.4)	19 (4.3)	26 (3.7)	
PDDS, n (%)^c^					0.001
Mild (0–1)	1417 (31.0)	1037 (30.0)	139 (31.7)	241 (35.2)	
Moderate (2–4)	1467 (32.1)	1083 (31.4)	151 (34.4)	233 (34.1)	
Severe (5–8)	1690 (36.9)	1331 (38.6)	149 (33.9)	210 (30.7)	
Number of comorbidities, n (%)					0.295
0	575 (12.4)	437 (12.5)	56 (12.5)	82 (11.8)	
1	903 (19.4)	685 (19.5)	70 (15.7)	148 (21.2)	
2	1062 (22.8)	793 (22.6)	102 (22.8)	167 (24.0)	
≥3	2108 (45.4)	1589 (45.3)	219 (49.0)	300 (43.0)	
Current smoker, n (%)	262 (5.6)	219 (6.3)	20 (4.5)	23 (3.3)	0.005
Alcohol intake, n (%)					<.001
Never	1716 (37.0)	1371 (39.1)	155 (34.8)	190 (27.3)	
Monthly or less	1211 (26.1)	900 (25.7)	118 (26.5)	193 (27.8)	
Two to four times per month	743 (16.0)	543 (15.5)	77 (17.3)	123 (17.7)	
Two to three times per week	483 (10.4)	352 (10.1)	52 (11.7)	79 (11.4)	
Any physical activity, n (%)	2678 (57.8)	1958 (56.0)	284 (63.8)	436 (62.7)	<.001

CIS: clinically isolated syndrome; PDDS: patient-determined disease steps.

a Nonresponders were defined as Fall 2023 survey participants who did not contact NARCOMS to express interest in the genetics substudy following the recruitment invitation.

For the concordance analyses investigating hypothetical willingness to share genetic information and actual consent by linkage to the Fall 2023 survey data, of the 1128 participants with available linked survey and genetic substudy data, 669 (59.3%) provided concordant responses and 459 (40.7%) discordant. Concordance comprised 586 participants who expressed hypothetical willingness and consented and 83 who expressed unwillingness and did not consent. Among discordant responses, 359 expressed willingness but did not consent, while 100 consented despite indicating unwillingness. In aggregate, 945 participants reported hypothetical willingness whereas 686 consented, and more than half of those indicating unwillingness (100 of 183) subsequently consented. Overall, the kappa statistics indicated low agreement between the genetic study response status and willingness to link genetic data (simple kappa = 0.0469, standard error = 0.0256; adjusted kappa = 0.1862, standard error = 0.0293). Whether participants’ responses to the Fall 2023 survey and to participation in the genetics substudy were concordant or not did not differ by demographics, apart from household income and national ADI (Table 2). Multivariable regression analyses identified no factors to be significantly associated with a concordant response between consent status and hypothetical willingness to share genetic information (Table 3).

**Table 2.** Characteristics associated with discordant responses between NARCOMS participants hypothetically willing to provide a saliva sample for genetic analyses and actual enrollment in genetic study.

Characteristics	Concordant (n = 669)	Discordant (n = 459)	*P*-value
Age at time of recruitment for genetic substudy			
Age at time of fall 2023 survey (years), mean (SD)	64.6 (9.42)	65.5 (9.77)	0.153
Age at MS symptom onset (years), mean (SD)	31.6 (10.13)	31.5 (9.46)	0.923
Age at MS diagnosis (years), mean (SD)	38.3 (9.83)	39.4 (9.55)	0.054
Disease duration (years), mean (SD)	25.4 (9.37)	25.0 (8.89)	0.466
Clinical course, n (%)			0.411
Clinically isolated syndrome/relapsing remitting	372 (56.5)	266 (59.8)	
Primary or secondary progressive	245 (37.2)	158 (35.5)	
Don’t know	41 (6.2)	21 (4.7)	
Female, n (%)	557 (83.3)	374 (81.5)	0.440
Race, n (%)			0.881
White	578 (86.4)	396 (86.3)	
Black	11 (1.6)	6 (1.3)	
Other	80 (12.0)	57 (12.4)	
Education at enrollment, n (%)			0.470
≤High school/GED	203 (30.9)	131 (28.9)	
≥Postsecondary	453 (69.1)	322 (71.1)	
Annual household income, n (%)			0.023
Less than $50,000	147 (22.0)	127 (27.8)	
$50,001–$100,000	210 (31.4)	135 (29.5)	
Over $100,000	223 (33.4)	122 (26.7)	
I do not wish to answer	88 (13.2)	73 (16.0)	
Any health insurance, n (%)	657 (98.2)	444 (96.7)	0.112
Marital status, n (%)			0.024
Single	179 (26.8)	151 (33.0)	
Partnered	489 (73.2)	306 (67.0)	
Employment status, n (%)			0.541
Full-time	135 (20.2)	87 (19.0)	
Part-time	77 (11.5)	45 (9.8)	
Not employed	457 (68.3)	326 (71.2)	
Rural-urban commuting area, n (%)			0.251
Urban-focused	572 (85.6)	384 (83.7)	
Large rural city/town-focused	55 (8.2)	35 (7.6)	
Small or isolated rural town-focused	41 (6.1)	40 (8.7)	
Area deprivation index, mean (SD)			
National rank	36.8 (24.34)	40.4 (24.39)	0.009
State rank	4.1 (2.62)	4.3 (2.65)	0.159
Area deprivation index, national rank (quantiles), n (%)			0.078
0–25	260 (39.2)	147 (32.3)	
26–50	221 (33.3)	157 (34.5)	
51–75	125 (18.9)	107 (23.5)	
76–100	57 (8.6)	44 (9.7)	
Source of care, n (%)			0.825
General practitioner	23 (3.4)	18 (3.9)	
Private neurologist	302 (45.3)	206 (45.1)	
Specialized MS center	287 (43.0)	199 (43.5)	
Veteran's Administration	23 (3.4)	10 (2.2)	
Other	9 (1.3)	5 (1.1)	
Do not receive care for MS	23 (3.4)	19 (4.2)	
PDDS, n (%) ^c^			0.355
Mild (0–1)	228 (34.8)	146 (32.4)	
Moderate (2–4)	230 (35.1)	151 (33.5)	
Severe (5–8)	197 (30.1)	154 (34.1)	
Number of comorbidities, n (%)			0.069
0	83 (12.4)	53 (11.6)	
1	144 (21.5)	72 (15.8)	
2	146 (21.8)	118 (25.8)	
≥3	296 (44.2)	214 (46.8)	
Current smoker, n (%)	22 (3.3)	21 (4.6)	0.267
Alcohol intake, n (%)			0.059
Never	186 (27.9)	153 (33.5)	
Monthly or less	182 (27.3)	124 (27.1)	
Two to four times per month	115 (17.2)	84 (18.4)	
Two to three times per week	80 (12.0)	49 (10.7)	
Any physical activity, n (%)	418 (62.9)	294 (64.3)	0.614

**Table 3.** Multivariable regression investigation of factors associated with discordant responses for NARCOMS participants who were hypothetically willing to provide a saliva sample for genetic analyses and not enrolling in genetic study.

Effect	Odds ratio estimate (95% confidence limits)	*P*-value
Age (present year), 10-year increments	0.882 (0.761, 1.021)	0.0931
Annual household income		
<$50,000	Reference	
$50,001–$99,999	1.250 (0.888, 1.760)	0.2016
$100,000 or more	1.286 (0.883, 1.873)	0.1900
I do not wish to answer	0.941 (0.622, 1.422)	0.7723
Marital status		
Single	Reference	
Partnered	1.179 (0.882, 1.576)	0.2656
Area deprivation index quartile		
0–25^th^ (lowest)	Reference	
26–50	0.836 (0.622, 1.125)	0.2369
51–75	0.703 (0.499, 0.991)	0.0445
76–100 (highest)	0.807 (0.512, 1.275)	0.3585
MS disease duration, 10-year increment	1.111 (0.957, 1.290)	0.1673
Has health insurance		
No	Reference	
Yes	0.543 (0.242, 1.218)	0.1387

Hosmer and Lemeshow Goodness-of-Fit Test: χ2 (df = 8) = 10.1; *P* = 0.26.

## Discussion

The costs of generating genetic data have dropped by orders of magnitude in the last 20 years^18^; their addition to long-term MS registries has the potential to identify genetic factors associated with disease outcomes. In this pilot study, we assessed the feasibility and acceptability of collecting saliva for genetic data generation linked to the US-based MS registry, NARCOMS, and whether responses to past hypothetical questions regarding sharing genetic information reflected the actual observed behavior. As hypothesized, we found between 62.2% and 73.2% of NARCOMS participants were willing to provide saliva samples for genetics studies. However, when considering all approximately 6200 NARCOMS participants sent an invitation to participate, the overall participation rate was ∼10%, reflecting the combined effect of nonresponse to initial contact, nonconsent, and nonreturn of saliva kits. This overall rate is important context for feasibility planning, and both metrics are relevant: conditional consent rates speak to acceptability among engaged participants, while the overall rate reflects the realistic yield of a passive recruitment approach in a registry setting. We also found that hypothetical sharing of genetic information did not reflect actual behaviors; and did not identify any demographic or clinical factors associated with higher odds of providing concordant responses.

Between 60% and 70% of NARCOMS participants consented to provide a saliva sample for genetics studies. This rate is in line with a past study on the genetics of postpartum depression which used a mobile app for recruitment.^11^ Saliva is known to be a valuable test material, given the easy availability, noninvasiveness, and low cost and has been broadly used for investigating genetics,^8^ and other etiological factors.^19^ Collected saliva was returned within a median of 17 days from sending the collection kit to the participant, which is similar to a Scottish population-based health study (Generation Scotland) finding the median time was 13 (interquartile range: 8–21) days.^20^ Studies in non-MS populations have found decreased probability of saliva kit return with increasing time between address confirmation and receipt of saliva collection kit.^11^

Recently, members of our group found >60% of 4980 NARCOMS registry participants would be willing to link their genetic data to support clinical trials.^10^ Whether this response would differ if the study were observational rather than a clinical trial is unknown. Like that study, we also found those with MS consenting to participate in the genetics substudy was greater among those with higher household income, which is also found in general for research participation.^21^ We found discordance between hypothetical willingness to share genetic information for a clinical trial and their actual consent to provide a saliva sample for genetic studies in academic institutions. Overall, 945 participants reported willingness while 686 ultimately consented, so stated willingness exceeded observed participation by ∼38%. The discordancy was not symmetric, as more than half of those expressing unwillingness nonetheless consented, indicating that the hypothetical items predicted subsequent behavior poorly in both directions. These findings suggest hypothetical willingness overestimates the pool of likely participants and should not be used as a proxy for actual participation when projecting recruitment, although a single registry cannot establish whether discordance introduces bias or affects sample representativeness. This discordance could be solely attributed to a lack of willingness to provide information specifically for a clinical trial for concerns on misuse or safety. While we were unable to identify any factors associated with discordancy and there draw any conclusions about differential representativeness across subgroups, this finding suggests future studies should consider supplementing hypothetical willingness estimates with pilot data from comparable populations.

Health literacy is high amongst NARCOMS participants.^22^ Education level, a proxy for health literacy, varied across participants categories: nonresponders had the lowest proportion with greater than postsecondary education (55.9%), while consented (68%) and declined (71%) participants were higher. Elevated education amongst decliners suggests health literacy may facilitate informed refusal as much as participation. Future studies should incorporate validated health literacy measures to better understand this relationship.

This study had strengths including a large sample of people with MS, with extensive data collected. This study was limited by voluntary nature of NARCOMS, and the potential lack of generalizability to younger people with MS or those of lower socioeconomic status, since few were represented. Importantly, the overall participation rate of approximately 10% relative to the eligible registry population reflects the yield of a passive, opt-in recruitment strategy as shown by studies in hereditary cancers or myocardial infarction.^23,24^ Future studies should anticipate this and include active outreach. Studies should also account for potential delays in mailing, and lost kits. The concordance analysis was restricted to participants who responded to a prior hypothetical willingness survey and were subsequently approached for the genetic substudy, representing a selected subset. Because all participants are enrolled in NARCOMS, this subset is not distinguished by a general willingness to engage in research; the more relevant consideration is whether survey responders differed systematically from nonresponders. In the originating survey, responders and nonresponders were broadly comparable with respect to age, sex, and educational attainment, suggesting demographic selection is unlikely to fully account for the observed level of concordance. Fewer than 5% of linked participants were excluded from the analytic sample due to incomplete responses. We cannot, however, exclude residual differences on unmeasured characteristics, and these estimates should be interpreted as applying to this responder population rather than the full eligible registry.

The hypothetical willingness question and genetic substudy recruitment were separated by approximately 1–2 years, during which participant attitudes toward genetic data sharing or personal circumstances may have changed. The adjusted kappa of 0.186 therefore likely reflects both the intention-behavior gap and temporal attitude change, which cannot be disentangled in the current data. Future studies should minimize this interval or reassess willingness closer to the point of recruitment.

In conclusion, among NARCOMS participants who were contacted and engaged, willingness to provide saliva samples for genetics studies was relatively high. However, the overall participation rate of approximately 10% across the eligible registry population underscores that passive recruitment strategies have real-world limits and findings from this single registry may not generalize to other MS registries or broader MS populations. Hypothetical sharing of genetic information did not reflect actual behaviors, but we were unable to identify any factors associated with higher odds of providing concordant responses. While these results offer a useful benchmark for planning future registry-based genetic studies, replication in other registries and with more active recruitment strategies will be needed before broad conclusions about feasibility can be drawn. Linking genetic data to existing MS registries with long-term follow-up will permit many future discoveries.
